# Cerebral Tau PET with ^18^F‐APN‐1607 in Alzheimer's disease: a multicenter phase III diagnostic study

**DOI:** 10.1002/alz70856_101593

**Published:** 2025-12-25

**Authors:** Mei Cui, Yu‐Yuan Huang, Yiren Fan, Jin‐Tai Yu

**Affiliations:** ^1^ MOE Frontiers Center for Brain Science, Fudan University, Shanghai, China; ^2^ Huashan Hospital, Fudan University, Shanghai, Shanghai, China; ^3^ Department of Neurology and National Center for Neurological Disorders, Huashan Hospital, State Key Laboratory of Medical Neurobiology and MOE Frontiers Center, Fudan University, Shanghai, Shanghai, China; ^4^ National Center for Neurological Disorders, Shanghai, China

## Abstract

**Background:**

Beta‐amyloid (Aβ) and tau are key pathological features of Alzheimer's disease (AD). ^18^F‐florzolotau (^18^F‐APN‐1607) was developed for high‐contrast imaging of tau fibrils in both AD and non‐AD conditions. Phase 1 and 2 trials have studied the pharmacokinetics, safety, and diagnostic efficacy of ^18^F‐APN‐1607. This phase III, multicenter trial demonstrates the sensitivity, specificity, and safety of ^18^F‐APN‐1607 in diagnosing MCI due to AD and AD, and highlights the correlation between tau pathology staging and clinical severity.

**Method:**

This study is a multicenter, Phase III clinical trial conducted at 11 tertiary medical centers in China. Participants included 30 healthy volunteers (HV), 100 MCI due to AD and 100 AD dementia. All participants meet the core clinical diagnostic criteria for AD dementia according to the 2018 NIA‐AA guidelines. The primary outcomes included: (1) Subjective Reading Analysis; (2) SUVR‐Based Reading Analysis, defining negative and positive cases and calculating sensitivity and specificity for tau deposition. Secondary objectives focused on the safety and tolerability of ^18^F‐APN‐1607. Exploratory endpoints: Measurement of ^18^F‐APN‐1607 SUVR based on Braak staging and evaluate of the correlation between SUVR in these ROIs and clinical severity.

**Result:**

A threshold based on SUVRs 2 SD above the mean binding in the three groups was utilized. The area under the ROC curve was 0.982 in the medial temporal lobe (MTL) region and 0.979 in the neocortex region when differentiating AD from HV. In contrast, when distinguishing MCI from HV, the area under the ROC curve was slightly lower, at 0.842 and 0.869 for the MTL and neocortex regions, respectively. No Serious Adverse Events (SAEs), TEAEs leading to dose reduction, interruption, or discontinuation of the study drug. ^18^F‐APN‐1607 PET imaging showed distinct patterns of tau accumulation across Braak stages I‐VI, with increasing SUVR values in individuals with MCI and AD. Robust correlations were observed between SUVR values and clinical and cognitive metrics, including CDR scores, MMSE scores, and ADAS‐cog scores.

**Conclusion:**

^18^F‐PM‐PBB3 has good specificity and sensitivity in diagnosing AD and is safe and reliable. It may also provide a reliable biomarker for tracking disease progression and cognitive decline in AD.

